# Validation of the questionnaire for impulsive-compulsive disorders in Parkinson’s disease (QUIP) and the QUIP-rating scale in a German speaking sample

**DOI:** 10.1007/s00415-014-7299-6

**Published:** 2014-03-09

**Authors:** Catharina Claudia Probst, Lina Marie Winter, Bettina Möller, Heinz Weber, Daniel Weintraub, Karsten Witt, Günther Deuschl, Regina Katzenschlager, Thilo van Eimeren

**Affiliations:** 1 Department of Neurology, Kiel University, Kiel, Germany; 2 Karl Landsteiner-Institut für Neuroimmunologische und Neurodegenerative Erkrankungen, Vienna, Austria; 3 Department of Neurology, Sozialmedizinisches Zentrum Ost, Donauspital, Vienna, Austria; 4 Department of Psychiatry, University of Pennsylvania, Philadelphia, USA

**Keywords:** ICD, Addiction, PD, QUIP, QUIP-RS

## Abstract

Impulsive-compulsive disorders are frequent in patients with Parkinson’s disease (PD). Recently, a screening questionnaire and rating scale were developed for these disorders: the questionnaire for impulsive-compulsive disorders (QUIP) and QUIP-rating scale (QUIP-RS). We assessed the validity of these instruments in the German language in order to reevaluate the benefit and to obtain German screening tools in clinical practice. A convenience sample of 156 patients was assessed in Kiel and Vienna. The patients filled out the QUIP-current, the QUIP-anytime and the QUIP-RS. We validated the questionnaires against a gold standard diagnosis via receiver operating characteristic curves and determined optimal cut-off scores for the instruments. Excluding walkabout, which was not shown to be valid, sensitivities ranged from 60–92 % for the QUIP-current, 68–91 % for the QUIP-anytime, and 73–100 % for the QUIP-RS. Specificities were >71 % for QUIP-current, >69 % for QUIP-anytime and >62 % for QUIP-RS. With its very good sensitivities, the QUIP-RS is a valid instrument to assess impulsive-compulsive disorders and makes an early detection of behavioral disorders in PD possible. The QUIP-anytime was also shown to be a valid screening instrument. Both are expected to prove useful in scientific and clinical practice.

**Electronic supplementary material:**

The online version of this article (doi:10.1007/s00415-014-7299-6) contains supplementary material, which is available to authorized users.

## Introduction

Within the last several years, impulsive-compulsive disorders have been recognized as side effects of dopamine replacement therapy in Parkinson’s disease (PD) [1]. The largest epidemiological study found impulse control disorders (ICDs: pathological gambling, hypersexuality, excessive buying or binge eating) in 14 % of medicated PD patients and 17 % of PD patients treated with dopamine agonists [2]. Aside from the ICDs, other repetitive, compulsive behaviors develop as a consequence of dopaminergic medication. Punding has been defined by repetition of simple motor actions, e.g. excessive and aimless sorting or manipulation of items [3]. Hobbyism is a more complex form of punding: patients are overly engaged with their hobbies and neglect other activities (e.g. hygiene) [3]. Sometimes, walkabout (excessive and aimless walking) is described as an independent disorder [4]. Furthermore, some patients develop dopamine dysregulation syndrome (DDS) which is characterized by an addiction-like use of dopaminergic medication [4].

Common features of the disorders are that patients perform specific behaviors excessively, eventually lose control over them and consequently have to face serious socio-economic consequences [3]. Early interventions, i.e. medication changes or psychotherapeutic support, effectively improve symptoms and can prevent severe consequences [5]. However, some patients have no insight or do not associate their behaviors with dopaminergic medication, and thus do not report them to the neurologist.

Until recently there was no questionnaire for efficient early detection of these disorders in PD patients [6]. Therefore, Weintraub et al. [7] developed the questionnaire for impulsive-compulsive disorders in Parkinson’s disease (QUIP) and the questionnaire for impulsive-compulsive disorders in Parkinson’s disease-rating scale (QUIP-RS) [6]. The QUIP screens for pathological gambling, hypersexuality, excessive buying, overeating, punding, hobbyism, walkabout and DDS. The QUIP-RS assesses the severity of the same behaviors (excluding walkabout) with a Likert scale.

The American validation of the questionnaires has provided satisfactory sensitivities and specificities [6, 7]. We performed an independent validation of the German versions of QUIP and QUIP-RS in order to add information to their clinimetric properties and to have quality criteria for use in the German language.

## Method

### Questionnaires

There are two versions, current and anytime, for the QUIP and a current version for the QUIP-RS. The current questionnaires assess the symptoms for the last 4 weeks, the anytime questionnaire asks about the 4 weeks with the worst symptoms since the beginning of PD. Apart from the time frame, the two QUIP versions are identical. The questionnaires validated in the present study are provided in the supplementary section (including scoring information).

### QUIP

A description of the questionnaire development is found in [7]. The QUIP was translated into German, retranslated into English by a native speaker and finally proof-read by the primary author of the original version (D.W.). The QUIP is a dichotomous questionnaire which can be filled out by the patient, with the help of a third person or by a third person alone.

For scoring, positive answers for each disorder are summed up leading to a maximum of five points for each of the ICDs and DDS, and three points for punding, hobbyism and walkabout.

### QUIP-RS

The QUIP-RS [6] was derived from the QUIP with the aim to create a rating scale that assesses severity and change of symptoms. Unlike the QUIP, walkabout is not listed as an independent disorder in the rating scale. The translation procedure was the same as for the QUIP. Additionally, eight PD patients and five healthy controls evaluated the questionnaire concerning readability. Subsequently, improvements in wording were made. Finally, a native speaker translated the QUIP-RS back into English and the primary author (D.W.) confirmed these changes.

The QUIP-RS consists of an instruction sheet and a second sheet with four questions which have to be answered for each disorder on a 5-point Likert scale. Scoring range for each scale (i.e. disorder) is 0–16. The QUIP-RS can be answered by the patient, with the help of a third person or by a third person alone.

### Diagnostic interview

In order to validate the questionnaires against a gold standard, we developed a semistructured diagnostic interview based on the procedure of the American validation [6]. We included the diagnostic statistical manual of mental disorders IV-TR criteria for binge eating and a structured clinical interview for pathological gambling [8, 9]. The criteria for binge eating were adapted to include episodes of general overeating [7]. For hypersexuality and excessive buying the currently proposed research criteria were used [3, 10]. The sections for punding, hobbyism, walkabout and DDS were based on common descriptions of the symptoms [1, 3, 4].

To exclude manic or hypomanic episodes as differential diagnoses we also assessed the corresponding symptoms [11]. Furthermore, we included the Structured Clinical Interview for DSM-IV Axis I disorders section for depressive/dysthymic symptoms [11].

The disorders were assessed for several time frames: (1) the time of the interview and the 4 weeks before filling out the QUIP-RS, (2) the 4 weeks before filling out the QUIP, (3) a time frame of at least 4 weeks with the worst symptoms during PD, (4) a time frame of at least 4 weeks with the worst symptoms before the beginning of PD.

For each of the disorders a severity index was determined:

0. Not present.
1. Subsyndromal, i.e. significant change in behavior or marked distress present but not all of A or B criteria are fulfilled (e.g. for pathological gambling, only four instead of five A-criteria are met; or for binge eating, only general overeating present).
2. Criteria met—weak manifestation.
3. Criteria met—moderate manifestation.
4. Criteria met—strong manifestation.

The manifestation level was determined by the subjective distress felt by the patient and/or the dimensions of consequences of the disorder.

### Patient sample and validation process

The sample consisted of 156 PD patients (106 male). The data were collected between October 2011 and July 2013 in two centers: Kiel (133 patients) and Vienna (23 patients). The study was approved by the local ethics committees and conducted according to the Declaration of Helsinki [12].

### Kiel sample

We sent a letter with study description, consent form and German versions of QUIP-current and QUIP-anytime to 1,207 PD patients of the Department of Neurology in Kiel. Patients were asked to fill out the questionnaires on their own or with a partner and return those together with the signed consent form. Mail was returned as undeliverable in 265 cases. Three hundred and eighty-seven patients (response rate 32.06 %) sent back completed forms. These returns were classified as symptomatic and non-symptomatic based on the American norms of the QUIP-current [7]. The final sample consisted of 65 positively and 65 negatively screened patients (matched for gender and age). They filled out the QUIP-RS in the study center and were subsequently interviewed. This selection process guaranteed an adequate number of positives for validation. Three more patients were recruited from the ward of the Department of Neurology.

Two psychologists, C.P. and B.M., conducted the interviews. They agreed upon a standardized application of the diagnostic criteria together with a psychiatrist (D.W.) and a neurologist (T.E.). The psychologists evaluated the first six patients together and were blinded to the outcome of the questionnaires, which guaranteed an independent gold standard diagnosis.

Thirteen patients of the Kiel sample filled out the questionnaires with help from a third person and were, therefore, interviewed with the respective partner. These data were included in the sample data and were not analyzed separately.

With the aim to assess test–retest reliability, 116 patients filled out the QUIP-RS a second time after a period of 11–18 days.

### Vienna sample

There were two minor differences in the validation procedure. In contrast to the Kiel sample, the 23 Austrian patients were directly recruited from in- or outpatients of the Department of Neurology of the Donauspital Wien. Moreover, patients filled out the questionnaires after the diagnostic interview.

### Analyses

The questionnaire scales were validated against the diagnoses of the interview. For the QUIP-RS, the diagnoses of time frame 1 were used and for the QUIP-current, the ones of time frame 2. For the QUIP-anytime, the time frames 2 and 3 were combined. In order to detect vulnerable patients as early as possible, subsyndromal cases were included in the calculation as positives.

Receiver operating characteristic (ROC) curves and area under the curves (AUC) were calculated for each scale. The final cut-off scores were defined using the Youden index [13], if sensitivities exceeded 0.7 and specificities were ≥0.6. Otherwise, we tried to find a cut-off with a sensitivity ≥0.7 and a specificity ≥0.6. This procedure was implemented in order to detect as many patients as possible but at the same time keeping the test efficient and meaningful by trying to keep false-positives low.

The retest reliabilities of the QUIP-RS were determined via ICC (2, 1) [14].

To explore if data quality differed between the two centers, ROC curves were calculated separately for Kiel and Vienna and were compared with Venkatraman’s test for two unpaired ROC curves [15].

In clinical practice, it may be useful to make the patient and the informant complete the questionnaires separately [16]. However, a separate analysis of the subgroup that filled out the questionnaires with the help of a partner could not be done in the present study due to the low number of these cases (*n* = 13).

Subjects with missing values were deleted in the calculation in a pairwise manner. SPSS Statistics 21.0 Software (IBM SPSS Inc. Chicago, IL) and GNU R statistics were used for the analysis.

## Results

### Sample characteristics

A sample description regarding age, PD duration, medication use, brain stimulation and depression can be found in Table 1. Six patients reported manic or hypomanic symptoms in one of the time frames but these symptoms did not coincide with any impulsive-compulsive disorder.

**Table 1:** Description of the patient groups with (positives) and without (negatives) at least subsyndromal disorders at the time of the interview and since the beginning of PD

	Age in years *M* (SD)	PD duration in years *M* (SD)	LEDD in mg *M* (SD)	LEDDA in mg *M* (SD)	Sex	DBS	Depressive/dysthymic symptoms
At the time of the interview							
Whole sample (*n* = 156)	63.12 (9.87)	9.46 (6.15)	869.95 (663.05)	166.43 (155.93)	*m* = 105 *f* = 51	26	35
Positives (*n* = 72)	61.94 (9.94)	9.08 (5.36)	980.00 (761.67)	167.91 (152.30)	*m* = 50 *f* = 22	10	26
Negatives (*n* = 84)	64.10 (9.76)	9.78 (6.77)	777.34 (555.08)	165.16 (159.89)	*m* = 55 *f* = 29	16	9
Significance (*t* test, Fisher’s exact test)	*p* = 0.09 (one-sided)	*p* = 0.47 (two-sided)	***p*** **=** **0.03 (one-sided)**	*p* = 0.45 (one-sided)	*p* = 0.60 (two-sided)	*p* = 0.41 (two-sided)	***p*** **<** **0.001 (one-sided)**
Since the beginning of PD							
Positives (*n* = 82)	61.51 (10.02)	9.51 (5.62)	958.73 (727.58)	163.31 (154.54)	*m* = 59 *f* = 23	13	42
Negatives (*n* = 74)	64.86 (9.46)	9.39 (7.72)	775.08 (576.29)	169.80 (158.41)	*m* = 46 *f* = 28	13	14
Significance (*t* test, Fisher’s exact test)	***p*** **=** **0.01 (one-sided)**	*p* = 0.89 (two-sided)	***p*** **=** **0.04 (one-sided)**	*p* = 0.40 (one-sided)	*p* = 0.23 (two-sided)	*p* = 0.83 (two-sided)	***p*** **<** **0.001 (one-sided)**

One-sided *p* values are used, if a hypothesis based on the current state of research was possible (ICD patients are younger, take higher medication dosages and have a higher prevalence of depression [2]). One-sided *p* values ≤0.05 and two-sided *p* values ≤0.1 were defined as significant (written in bold)

*M* mean, *SD* standard deviation, *LEDD* Levodopa daily equivalence dose, *LEDDA* dopamine agonist equivalence dose (calculated as in [17]), *DBS* deep brain stimulation

**Fig. 1:**
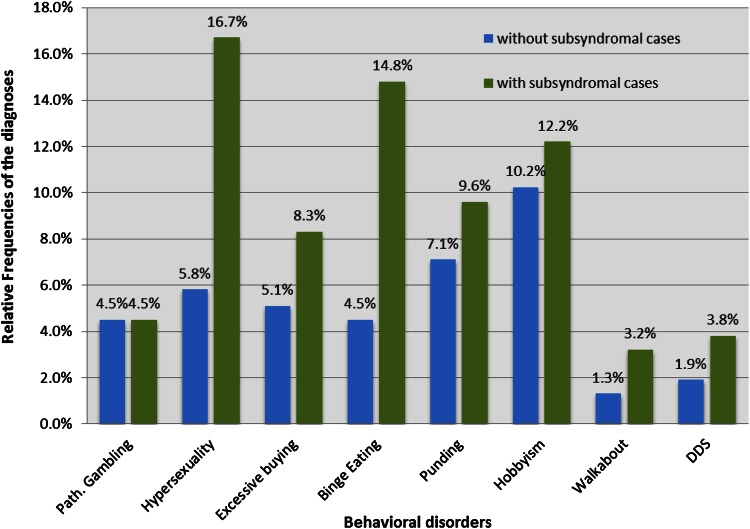
Relative frequencies of the impulsive-compulsive disorders at the time of the interview

At the time of the interview, 33 patients were diagnosed with at least one impulsive-compulsive disorder. Including the subsyndromal cases, 72 patients were classified as symptomatic via the diagnostic interview. Figure 1 displays the relative frequencies of the individual disorders. Twenty-five patients with at least subsyndromal symptoms had more than one diagnosis at the time of the interview. Note: our patient sample was preselected. Therefore, the frequencies of the disorders are not representative.

Over the course of PD, 52 patients had at least one behavioral disorder (82 including subsyndromal cases). The diagnoses (including subsyndromal cases) are as follows: pathological gambling, 9.0 % (9.0 %); hypersexuality, 14.7 % (23.7 %); excessive buying, 9.0 % (12.8 %); binge eating, 10.9 % (20.5 %); punding, 6.4 % (11.5 %); hobbyism, 10.3 % (17.3 %); walkabout, 3.2 % (4.5 %); and DDS, 8.3 % (9.0 %).

### QUIP-current

One hundred and fifty-six patients filled out the QUIP-current. AUC, the optimal cut-off scores, corresponding sensitivities and specificities are seen in Table 2. Four scales showed sensitivities below 0.7, but all specificities were ≥0.71. Still, the scales for walkabout and DDS did not reach a significant AUC at all.

**Table 2:** AUC as well as sensitivities and specificities for the chosen cut-off scores for the QUIP-current scales

	*n*	AUC	Cut-off	Sensitivity	Specificity
Gambling	153	0.84**	1	0.71	0.93
Sex	152	0.73***	1	*0.67*	0.80
Buying	153	0.92***	1	0.92	0.87
Eating	152	0.83***	1	0.76	0.84
Punding	154	0.75**	1	0.71	0.81
Hobbyism	151	0.71**	1	*0.67*	0.71
Walkabout	155	0.65	1	*0.40*	0.90
DDS	155	0.74	1	*0.60*	0.84

*n* subjects without missing values for the respective scale,* italics* sensitivities and specificities <0.70

** *p* < 0.01, *** *p* < 0.001

There were no significant differences between the ROC-curves of the Kiel and Vienna samples (*p* values between 0.33 and 0.99).

### QUIP-anytime

The QUIP-anytime was completed by 150 subjects. Table 3 shows the optimal cut-offs and corresponding sensitivities, specificities and AUC. The punding and walkabout scales had sensitivity values <0.7, but the remaining scales reached sensitivities ≥0.76 and all scales showed specificities ≥0.7. The AUC of the walkabout scale was not significantly different from 0.5.

**Table 3:** AUC as well as sensitivities and specificities for the chosen cut-off scores for the QUIP-anytime scales

	*n*	AUC	Cut-off	Sensitivity	Specificity
Gambling	141	0.91***	3	0.83	0.98
Sex	142	0.79***	1	0.75	0.79
Buying	139	0.90***	1	0.86	0.90
Eating	139	0.85***	1	0.78	0.88
Punding	149	0.75***	1	*0.69*	0.81
Hobbyism	148	0.76***	1	0.78	0.70
Walkabout	148	0.59	1	*0.25*	0.92
DDS	148	0.92***	1	0.91	0.86

*n* subjects without missing values for the respective scale,* italics* sensitivities and specificities <0.70

** *p* < 0.01, *** *p* < 0.001

The separately calculated ROC curves for the two centers did not differ significantly for any of the scales (*p* values between 0.14 and 0.72).

### QUIP-RS

#### Validity

One hundred and fifty-four patients filled out the QUIP-RS. In Table 4 the optimal cut-off scores as well as corresponding sensitivities, specificities and AUC are listed. All sensitivities reached values ≥0.73; only two specificities fell below 0.70.

**Table 4:** AUC as well as sensitivities and specificities for the chosen cut-off scores for the QUIP-RS scales

	*n*	AUC	Cut-off	Sensitivity	Specificity
Gambling	153	0.89***	3	0.83	0.92
Sex	152	0.82***	5^a^	0.73	0.76
Buying	153	0.91***	5	0.83	0.88
Eating	147	0.89***	4	0.90	0.72
Punding	151	0.79***	3^a^	0.79	*0.65*
Hobbyism	149	0.76***	4	0.94	*0.62*
DDS	153	0.86**	3	1	0.70

*n* subjects without missing values for the respective scale,* italics* sensitivities and specificities <0.70

** *p* < 0.01, *** *p* < 0.001

^a^Not determined via Youden index

**Fig. 2:**
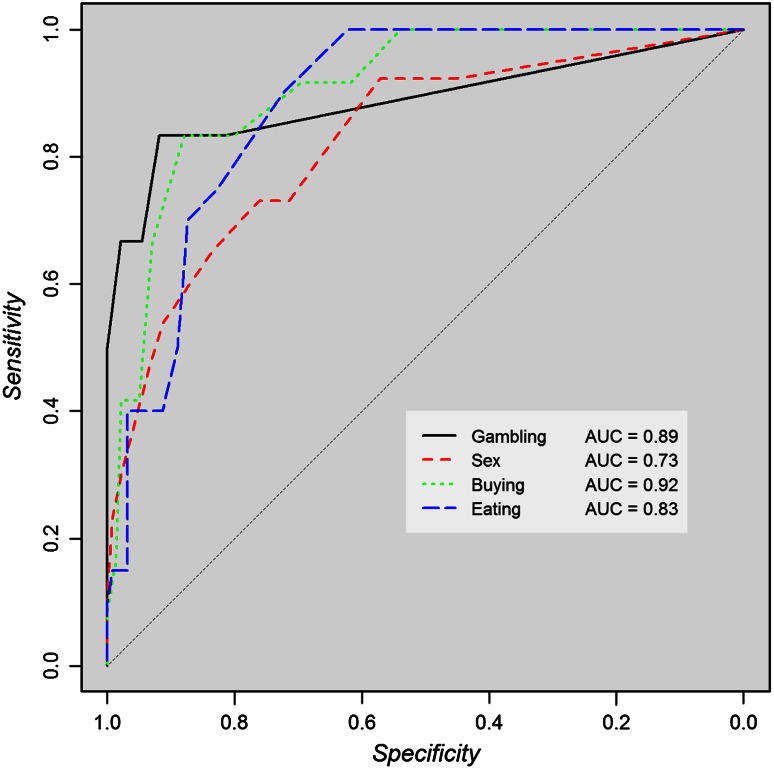
ROC curves of the impulse control disorder scales of the QUIP-RS

In contrast to the American validation, the validity of the punding and hobbyism scales did not improve by merging these scales (an AUC of 0.75 vs. 0.79 and 0.76). Therefore, we kept them as two separate scales. For hypersexuality and punding, the cut-off was determined in deviation from the Youden index. Figure 2 shows the ROC curves for the ICDs scales.

Means and standard deviations of the QUIP-RS scores can be found in the supplementary table (Supplementary 4).

Again, the comparison between the Vienna and Kiel ROC curves did not reveal any significant differences (*p* values between 0.23 and 1).

### Test–retest reliability

Twenty-seven questionnaires showed missing values in at least one scale. The ICC reliabilities were as follows: pathological gambling, *r* = 0.73; sex, *r* = 0.80; excessive buying, *r* = 0.71; binge-eating, *r* = 0.78; punding, *r* = 0.64; hobbyism, *r* = 0.57; DDS, *r* = 0.61; and for the entire QUIP-RS (sum score), *r* = 0.78.

## Discussion

The aim of the present study was to validate independently the QUIP-current, QUIP-anytime and the QUIP-RS and to obtain quality criteria for the German versions. In German speaking countries it is now possible to detect impulsive-compulsive disorders at an early stage and initiate interventions to prevent further consequences. The questionnaires also constitute necessary tools for scientific use.

Sensitivities and specificities of the QUIP-current proved to be satisfactory for pathological gambling, buying, eating and punding. Moderate sensitivities from 0.60 to 0.67 were found for the scales sex, hobbyism and DDS. The QUIP-anytime showed very good sensitivities and specificities (from 0.70 to 0.98) for the ICDs and DDS. With a value of 0.69, the sensitivity for punding was slightly lower. Walkabout has not turned out to be a valid scale for the QUIP questionnaires.

For the QUIP-RS, we found cut-offs with very good sensitivities (between 0.73 and 1) for all of the scales. Specificities for hobbyism and punding were moderate with values of 0.62 and 0.65. Compared to low sensitivities (i.e. a high false-negative rate), low specificities (i.e. a high false-positive rate) can be tolerated in a screening instrument, since false-positive patients may be at increased risk of developing a disorder in the future. The retest-reliabilities of the QUIP-RS can be considered satisfactory for the ICD scales and the entire questionnaire (*r* ranging from 0.71 to 0.80). For the remaining scales we only found moderate reliabilities (*r* between 0.57 and 0.64).

In the long run, the QUIP-RS will probably prove more useful than the QUIP-current because of better sensitivity, good retest reliability, and the possibility to assess symptom severity. Especially with regard to the detection of DDS, the QUIP-RS appears to be the better instrument since the AUC for this disorder is not significant in the QUIP. Albeit, we have to acknowledge that the low number of diagnoses (6) for this disorder makes the data vulnerable to biases in all three of the questionnaires. In any case, in clinical practice, a more detailed examination should be done to confirm the preliminary classification of the questionnaires.

The sensitivities and specificities found in the present study are not as good as the ones found by Weintraub et al. [7] for QUIP and QUIP-RS [6]. This might be due to linguistic differences occurring after the translation or a diverging distribution of the diagnoses. Also, differences in the validation procedure may have led to differing results. Our sample was mostly recruited via mail, whereas in the American validation, the patients were included in the context of routine clinical care. Furthermore, in the present study the QUIP-current was completed at home, in the US, on the contrary, it was filled out in the study center.

Our findings show that the ICD scales generally have good validity values, whereas the remaining scales are limited in their validity. Walkabout could not be assessed with the QUIP at all. Also, in the American validation of the QUIP, walkabout and punding showed lower sensitivities than the other scales. Low or moderate values for these scales might stem from the lack of commonly accepted criteria for these disorders and from the fact that patients do not always have insight into their behaviors [18]. These conditions may lead to a less valid and more error-prone assessment. Future research should focus on a more detailed symptom description and better defined diagnostic criteria for these disorders.

A limitation of the present study is that we did not systematically screen for cognitive deficits. However, all participants were able to follow the instructions and answer the questions adequately. Therefore, we can assume that demented patients were implicitly excluded from the study. For this patient group, the questionnaires do not constitute suitable screening instruments.

Future studies will need to investigate the sensitivity of the QUIP-RS to symptom changes after therapeutic interventions. With validations of the QUIP questionnaires in further languages and other patient samples taking dopamine agonist therapy, standardized screening and research tools will be available for use in international multicenter studies.

## Electronic supplementary material

Below is the link to the electronic supplementary material.

Supplementary 1a: German version of QUIP-current.

Supplementary 1b: German QUIP-current scoring sheet.

Supplementary 2a: German version of QUIP-anytime.

Supplementary 2b: German QUIP-anytime scoring sheet.

Supplementary 3a: German version of QUIP-RS.

Supplementary 3b: German QUIP-RS scoring sheet.

Supplementary 4: Means and Standard Deviations of the QUIP-RS scales for the whole sample and patients with and without at least subsyndromal disorders.

Supplementary material 1 (PDF 12 kb)

Supplementary material 2 (PDF 15 kb)

Supplementary material 3 (PDF 12 kb)

Supplementary material 4 (PDF 15 kb)

Supplementary material 5 (PDF 78 kb)

Supplementary material 6 (PDF 137 kb)

Supplementary material 7 (PDF 407 kb)
