# Supplementary material for: Validation of the questionnaire for impulsive-compulsive disorders in Parkinson’s disease (QUIP) and the QUIP-rating scale in a German speaking sample
Source: J Neurol. 2014 Mar 9;261(5):936–42. doi: 10.1007/s00415-014-7299-6 (PMC4148320; doi:10.1007/s00415-014-7299-6)
Supplement: Supplementary file 3 — Supplementary material 3 (PDF 12 kb) [file 415_2014_7299_MOESM3_ESM.pdf]

Fragebogen für impulsiv-zwanghafte Störungen bei der Parkinson-Krankheit (QUIP-IRGENDWANN)

Angaben durch: _____ Patient _____ Pflegeperson* _____ Patient und Pflegeperson

Patienten-ID:

Datum: ____

*Wenn Sie als Pflegeperson Angaben über einen Patienten machen, beantworten Sie die Fragen bitte auf der Basis Ihres Verständnisses des Patienten.

Beantworten Sie <u>ALLE FRAGEN</u> in Bezug auf Verhaltensweisen, die Sie <u>IRGENDWANN</u> während Ihrer Parkinsonerkrankung <u>FÜR MINDESTENS 4 WOCHEN</u> bei sich beobachtet haben.

<u>A. IMPULSKONTROLLSTÖRUNGEN</u>

1. Glauben Sie oder andere, dass Sie ein Problem mit übermäßigem Glücksspiel, Sex, Kaufen oder Essen haben? <u>Bitte beantworten Sie die Frage getrennt für alle vier Tätigkeiten</u>.

<u>Glücksspiel</u> (z. B. Casinos, Internet-Glücksspiele, Lotterien, Rubbellose, Wetten oder Spiel- oder Pokerautomaten) **__Ja __Nein**

<u>Sex</u> (z. B. unangemessene sexuelle Forderungen an andere stellen, häufig wechselnde Partner, Prostitution, Änderung der sexuellen Ausrichtung, Selbstbefriedigung, Internet- oder Telefonsex oder Pornos) **__Ja __Nein**

<u>Kaufen</u> (z. B. zu viele gleiche Dinge oder Dinge, die Sie gar nicht benötigen oder verwenden) **__Ja __Nein**

<u>Essen</u> (Essen Sie beispielsweise größere Mengen oder andere Nahrung als früher, schneller als normal, oder bis Sie ein unangenehmes Völlegefühl verspüren oder wenn Sie eigentlich gar nicht hungrig sind?) **__Ja __Nein**

2. Denken Sie zu häufig an die folgenden Tätigkeiten? (Haben Sie beispielsweise Probleme, bestimmte Gedanken aus dem Kopf zu bekommen, oder Schuldgefühle?)

**Glücksspiel**	**____Ja**	**____ Nein**
**Sex**	**____Ja**	**____ Nein**
**Kaufen**	**____Ja**	**____ Nein**
**Essen**	**____Ja**	**____ Nein**

3. Haben Sie das Gefühl, dass Ihr Drang oder Ihr Verlangen nach den folgenden Tätigkeiten übermäßig groß ist, oder ruft es bei Ihnen ein Gefühl der Verzweiflung hervor? (Fühlen Sie sich beispielsweise ruhelos oder gereizt, wenn Sie Ihrem Verlangen nicht nachgeben können?)

**Glücksspiel**	**____Ja**	**____ Nein**
**Sex**	**____Ja**	**____ Nein**
**Kaufen**	**____Ja**	**____ Nein**
**Essen**	**____Ja**	**____ Nein**

4. Fällt es Ihnen schwer, Ihr Verhalten in Bezug auf die folgenden Tätigkeiten zu kontrollieren? (Müssen Sie sich ihnen beispielsweise immer mehr widmen oder schaffen Sie es nicht, sich weniger intensiv damit zu beschäftigen oder ganz damit aufzuhören?)

**Glücksspiel**	**____Ja**	**____ Nein**
**Sex**	**____Ja**	**____ Nein**
**Kaufen**	**____Ja**	**____ Nein**
**Essen**	**____Ja**	**____ Nein**

Copyright © University of Pennsylvania 2008

Fragebogen für impulsiv-zwanghafte Störungen bei der Parkinson-Krankheit (QUIP-IRGENDWANN)

5. Kommt es vor, dass Sie ganz gezielt bestimmte Dinge tun, nur um sich weiterhin einer der folgenden Tätigkeiten hingeben zu können? (Versuchen Sie beispielsweise, Ihr Tun vor anderen zu verheimlich, lügen Sie oder horten Sie Dinge? Leihen Sie sich Geld von anderen? Machen Sie Schulden oder stehlen Sie? Beteiligen Sie sich an Straftaten?)

**Glücksspiel**	**____Ja**	**____ Nein**
**Sex**	**____Ja**	**____ Nein**
**Kaufen**	**____Ja**	**____ Nein**
**Essen**	**____Ja**	**____ Nein**

B. SONSTIGE VERHALTENSWEISEN

1. Glauben Sie oder andere, dass Sie zu viel Zeit damit verbringen,

A. sich bestimmten Aufgaben, Hobbys oder anderen, organisierten Aktivitäten (z. B. Schreiben, Malen, Gartenarbeit, dem Reparieren, Zerlegen oder Sammeln von Dingen, dem Computer oder der Projektarbeit) zu widmen? **__Ja __Nein**

B. bestimmte einfache Bewegungsabläufe (z. B. das Reinigen, Aufräumen, Hantieren, Untersuchen, Sortieren, Ordnen oder Anordnen von Gegenständen) zu wiederholen? **__Ja __Nein**

C. sinn- und ziellos in der Gegend herumzulaufen oder herumzufahren? **__Ja __Nein**

2. Glauben Sie oder andere, dass es Ihnen schwer fällt, die auf diese Tätigkeiten verwendete Zeit zu kontrollieren? **__Ja __Nein**

3. Beeinträchtigen diese Tätigkeiten Ihr tägliches Leben oder verursachen sie Schwierigkeiten in Beziehungen oder bei der Arbeit? **__Ja __Nein**

C. MEDIKAMENTENGEBRAUCH

1. Glauben Sie oder andere (einschließlich Ihrer Ärzte), dass Sie ständig zu viel von Ihren Parkinson-Medikamenten nehmen? **__Ja __Nein**

2. Haben Sie im Laufe der Zeit von sich aus und ohne ärztliche Anweisung Ihre gesamte Einnahme der Parkinson-Medikamente aus psychologischen Gründen erhöht, um beispielsweise Ihre Stimmung oder Motivation zu verbessern? **__Ja __Nein**

3. Haben Sie im Laufe der Zeit von sich aus und ohne ärztliche Anweisung mehr von Ihren Parkinson-Medikamenten eingenommen, weil Sie sich nur dann im Vollbesitz Ihrer körperlichen und geistigen Kräfte fühlen, wenn Sie Medikamente nehmen, auch wenn diese Dyskinesien (Überbewegungen) auslösen? **__Ja __Nein**

4. Fällt es Ihnen schwer, die Einnahme Ihrer Parkinson-Medikamente zu kontrollieren (z. B. weil Sie starkes Verlangen nach einer höheren Dosis empfinden oder weil Ihre Stimmung und Motivation schlechter sind bei einer geringeren Dosis)? **__Ja __Nein**

5. Horten oder verstecken Sie Ihre Parkinson-Medikamente, um die Gesamtdosis zu erhöhen? **__Ja __Nein**

Copyright © University of Pennsylvania 2008
