# Supplementary material for: Validation of the questionnaire for impulsive-compulsive disorders in Parkinson’s disease (QUIP) and the QUIP-rating scale in a German speaking sample
Source: J Neurol. 2014 Mar 9;261(5):936–42. doi: 10.1007/s00415-014-7299-6 (PMC4148320; doi:10.1007/s00415-014-7299-6)
Supplement: Supplementary file 4 — Supplementary material 4 (PDF 15 kb) [file 415_2014_7299_MOESM4_ESM.pdf]

Name: ________________________ Datum: ________________________

Fragebogen für impulsiv-zwanghafte Störungen bei der Parkinson-Krankheit (QUIP-irgendwann)

Auswertung

- Für den Skalensummenwert werden die positiven Antworten eines Störungsbildes aufsummiert.
- Bei B. SONSTIGE VERHALTENSWEISEN bezieht sich die Frage B.1.A auf Hobbyismus, B.1.B auf Punding und B.1.C auf Walkabout. Darauf folgen zwei allgemeine Fragen (B.2 und B.3), die sich auf alle drei dieser Verhaltensweisen beziehen. Zu einer positiven Antwort bei B.1.A-B.1.C werden die positiven Antworten bei B.2 und B.3 hinzugezählt. Somit können bei jeder Verhaltensweise unter B. SONSTIGE VERHALTENSWEISEN maximal drei Punkte erreicht werden.

**Skala**	**Erreichter Wert**	**Cut-Off-Wert**	**Klassifizierung**	
Glücksspiel	/5	≥3	O **auffällig**	O **unauffällig**
Sex	/5	≥1	O **auffällig**	O **unauffällig**
Kaufen	/5	≥1	O **auffällig**	O **unauffällig**
Essen	/5	≥1	O **auffällig**	O **unauffällig**
Hobbyismus	/3	≥1	O **auffällig**	O **unauffällig**
Punding	/3	≥1	O **auffällig**	O **unauffällig**
Walkabout	/3	--	--	--
DDS (Medikamente)	/5	≥1	O **auffällig**	O **unauffällig**

QUIP-RATING SCALE
Copyright © University of Pennsylvania 2009
