# Supplementary material for: Validation of the questionnaire for impulsive-compulsive disorders in Parkinson’s disease (QUIP) and the QUIP-rating scale in a German speaking sample
Source: J Neurol. 2014 Mar 9;261(5):936–42. doi: 10.1007/s00415-014-7299-6 (PMC4148320; doi:10.1007/s00415-014-7299-6)
Supplement: Supplementary file 5 — Supplementary material 5 (PDF 78 kb) [file 415_2014_7299_MOESM5_ESM.pdf]

Fragebogen für Impulskontrollstörungen bei Parkinson-Krankheit – Bewertungsskala

Kurzanweisung

Bitte lesen Sie die Kurzanweisung vor dem Ausfüllen des Fragebogens!
Sie finden hier Informationen zum relevanten Zeitrahmen, Erklärungen zu den im Fragebogen vorkommenden Verhaltensweisen sowie Erläuterungen zu den Häufigkeiten der Symptome.

ZEITRAHMEN
Beziehen Sie sich bei der Beantwortung der Fragen bitte auf die letzten vier Wochen.

BESCHREIBUNG DER VERHALTENSWEISEN
A. Glücksspiel (Casinos, Internet-Glücksspiele, Lotto, Rubbellose, Wetten, Spielautomaten oder Pokerautomaten)

B. Sex (unangemessene sexuelle Forderungen an andere stellen, wechselnde Sexualpartner, Prostitution, Veränderung der sexuellen Orientierung, Selbstbefriedigung, Internet-, Telefonsex oder Pornografie)

C. Kaufen (zu viel von einer Sache bzw. Dinge kaufen, die Sie nicht benötigen oder verwenden)

D. Essen (Essen von größeren Mengen oder anderen Nahrungsmitteln als früher, schneller als normal, zu anderen Zeiten (beispielsweise mitten in der Nacht), essen bis Sie sich unangenehm voll fühlen oder obwohl Sie gar nicht hungrig sind)

E. Hobbyismus (bestimmte Aufgaben, Hobbys oder andere geplante Tätigkeiten, wie z.B. Schreiben, Malen, Gartenarbeit, das Reparieren oder Montieren von Dingen, Computerbenutzung, Projektarbeit etc.)

F. Punding (Wiederholen von einfachen motorischen Tätigkeiten, wie z.B. Putzen, Aufräumen, Hantieren, Untersuchen, Sortieren, Sammeln, Ordnen oder Anordnen von Dingen etc.)

G. Medikamentengebrauch (immer wieder zu viel von den Parkinson-Medikamenten nehmen oder selbstständiges Erhöhen der Einnahme Ihrer Parkinson-Medikamente, ohne ärztliches Anraten)

HÄUFIGKEIT DER SYMPTOME

Nie (0)	= überhaupt nicht
Selten (1)	= kaum bzw. an einem Tag pro Woche
Manchmal (2)	= gelegentlich bzw. an 2-3 Tagen pro Woche
Oft (3)	= meistens bzw. an 4-5 Tagen pro Woche
Sehr oft (4)	= fast immer bzw. an 6-7 Tagen pro Woche

Copyright © University of Pennsylvania 2009

Fragebogen für Impulskontrollstörungen bei der Parkinson-Krankheit – Bewertungsskala

Berichtet von: _____ Patient _____ Angehöriger/Pflegekraft _____ Patient und Angehöriger

Patient / Proband: ____

Datum: ____

1. Wie oft denken Sie über die folgenden Verhaltensweisen nach (Haben Sie bspw. Schwierigkeiten, die Gedanken darüber aus dem Kopf zu bekommen oder haben Sie Schuldgefühle)?

A. Glücksspiele?	___Nie(0)	___Selten(1)	___Manchmal(2)	___Oft(3)	___Sehr oft(4)
B. Sex?	___Nie(0)	___Selten(1)	___Manchmal(2)	___Oft(3)	___Sehr oft(4)
C. Kaufen?	___Nie(0)	___Selten(1)	___Manchmal(2)	___Oft(3)	___Sehr oft(4)
D. Essen?	___Nie(0)	___Selten(1)	___Manchmal(2)	___Oft(3)	___Sehr oft(4)
E. Aufgaben durchführen / Hobbies nachgehen?	___Nie(0)	___Selten(1)	___Manchmal(2)	___Oft(3)	___Sehr oft(4)
F. Einfache Aktivitäten wiederholen?	___Nie(0)	___Selten(1)	___Manchmal(2)	___Oft(3)	___Sehr oft(4)
G. Ihre Parkinson-Medikamente nehmen?	___Nie(0)	___Selten(1)	___Manchmal(2)	___Oft(3)	___Sehr oft(4)

2. Haben Sie einen Drang nach den folgenden Verhaltensweisen, den Sie für zu exzessiv halten oder unter dem Sie leiden? Verspüren Sie bspw. Ruhelosigkeit oder Reizbarkeit, wenn Sie die Verhaltensweisen nicht durchführen können?

A. Glücksspiele?	___Nie(0)	___Selten(1)	___Manchmal(2)	___Oft(3)	___Sehr oft(4)
B. Sex?	___Nie(0)	___Selten(1)	___Manchmal(2)	___Oft(3)	___Sehr oft(4)
C. Kaufen?	___Nie(0)	___Selten(1)	___Manchmal(2)	___Oft(3)	___Sehr oft(4)
D. Essen?	___Nie(0)	___Selten(1)	___Manchmal(2)	___Oft(3)	___Sehr oft(4)
E. Aufgaben durchführen / Hobbies nachgehen?	___Nie(0)	___Selten(1)	___Manchmal(2)	___Oft(3)	___Sehr oft(4)
F. Einfache Aktivitäten wiederholen?	___Nie(0)	___Selten(1)	___Manchmal(2)	___Oft(3)	___Sehr oft(4)
G. Ihre Parkinson-Medikamente nehmen?	___Nie(0)	___Selten(1)	___Manchmal(2)	___Oft(3)	___Sehr oft(4)

3. Haben Sie Schwierigkeiten, die folgenden Verhaltensweisen zu kontrollieren (nehmen diese z.B. über die Zeit hinweg zu oder haben Sie Schwierigkeiten sie einzuschränken oder einzustellen)?

A. Glücksspiele?	___Nie(0)	___Selten(1)	___Manchmal(2)	___Oft(3)	___Sehr oft(4)
B. Sex?	___Nie(0)	___Selten(1)	___Manchmal(2)	___Oft(3)	___Sehr oft(4)
C. Kaufen?	___Nie(0)	___Selten(1)	___Manchmal(2)	___Oft(3)	___Sehr oft(4)
D. Essen?	___Nie(0)	___Selten(1)	___Manchmal(2)	___Oft(3)	___Sehr oft(4)
E. Aufgaben durchführen / Hobbies nachgehen?	___Nie(0)	___Selten(1)	___Manchmal(2)	___Oft(3)	___Sehr oft(4)
F. Einfache Aktivitäten wiederholen?	___Nie(0)	___Selten(1)	___Manchmal(2)	___Oft(3)	___Sehr oft(4)
G. Ihre Parkinson-Medikamente nehmen?	___Nie(0)	___Selten(1)	___Manchmal(2)	___Oft(3)	___Sehr oft(4)

4. Unternehmen Sie bestimmte Tätigkeiten (wie z.B. verbergen, was Sie tun, lügen, Dinge horten, von anderen etwas ausleihen, Schulden anhäufen, stehlen oder sich an illegalen Handlungen beteiligen), nur um die folgenden Verhaltensweisen fortsetzen zu können?

A. Glücksspiele?	___Nie(0)	___Selten(1)	___Manchmal(2)	___Oft(3)	___Sehr oft(4)
B. Sex?	___Nie(0)	___Selten(1)	___Manchmal(2)	___Oft(3)	___Sehr oft(4)
C. Kaufen?	___Nie(0)	___Selten(1)	___Manchmal(2)	___Oft(3)	___Sehr oft(4)
D. Essen?	___Nie(0)	___Selten(1)	___Manchmal(2)	___Oft(3)	___Sehr oft(4)
E. Aufgaben durchführen / Hobbies nachgehen?	___Nie(0)	___Selten(1)	___Manchmal(2)	___Oft(3)	___Sehr oft(4)
F. Einfache Aktivitäten wiederholen?	___Nie(0)	___Selten(1)	___Manchmal(2)	___Oft(3)	___Sehr oft(4)
G. Ihre Parkinson-Medikamente nehmen?	___Nie(0)	___Selten(1)	___Manchmal(2)	___Oft(3)	___Sehr oft(4)

Copyright © University of Pennsylvania 2009
