# Supplementary material for: Validation of the questionnaire for impulsive-compulsive disorders in Parkinson’s disease (QUIP) and the QUIP-rating scale in a German speaking sample
Source: J Neurol. 2014 Mar 9;261(5):936–42. doi: 10.1007/s00415-014-7299-6 (PMC4148320; doi:10.1007/s00415-014-7299-6)
Supplement: Supplementary file 6 — Supplementary material 6 (PDF 137 kb) [file 415_2014_7299_MOESM6_ESM.pdf]

Name: ________________________   Datum: ________________________

Fragebogen für Impulskontrollstörungen bei der Parkinson-Krankheit – Bewertungsskala

Auswertung

Skala	Erreichter Wert	Cut-Off-Wert	Klassifizierung	
A. Glücksspiel	/16	≥3	O **auffällig**	O **unauffällig**
B. Sex	/16	≥5	O **auffällig**	O **unauffällig**
C. Kaufen	/16	≥5	O **auffällig**	O **unauffällig**
D. Essen	/16	≥4	O **auffällig**	O **unauffällig**
E. Hobbyismus	/16	≥3	O **auffällig**	O **unauffällig**
F. Punding	/16	≥4	O **auffällig**	O **unauffällig**
G. DDS (Medikamente)	/16	≥3	O **auffällig**	O **unauffällig**

IKS Gesamtwert (A-D) ______ **(0-64)**

QUIP-RS Gesamtwert (A-G) ______ **(0-112)**

QUIP-RATING SCALE
Copyright © University of Pennsylvania 2009
