# Supplementary material for: Validation of the questionnaire for impulsive-compulsive disorders in Parkinson’s disease (QUIP) and the QUIP-rating scale in a German speaking sample
Source: J Neurol. 2014 Mar 9;261(5):936–42. doi: 10.1007/s00415-014-7299-6 (PMC4148320; doi:10.1007/s00415-014-7299-6)
Supplement: Supplementary file 7 — Supplementary material 7 (PDF 407 kb) [file 415_2014_7299_MOESM7_ESM.pdf]

Supplementary table Means and standard deviations of the QUIP-RS scales for the whole sample and patients with and without at least subsyndromal disorders.

	Whole sample				***Negatives***			***Positives***		
	n	Range	*M*	*SD*	*n*	M_0	SD_0	*n*	M_1	SD_1
**Gambling**	153	0-16	0.79	2.21	147	0.59	1.33	6	7.83	5.85
**Sex**	152	0-12	2.97	3.40	126	2.21	2.77	26	6.69	3.76
**Buying**	153	0-12	2.05	2.75	141	1.65	2.32	12	6.75	3.13
**Eating**	147	0-16	2.95	3.47	127	2.24	2.87	20	7.45	3.65
**Punding**	151	0-16	2.71	3.55	137	2.36	3.33	14	6.14	4.00
**Hobbyism**	149	0-16	3.61	3.84	133	3.23	3.66	16	6.81	3.87
**DDS**	153	0-16	2.72	4.06	146	2.50	3.88	6	8.33	4.97
**Sum score**	138	0-96	17.25	17.10	81	12.21	15.06	57	24.42	17.39

n: subjects with no missing values for the respective scale; M, SD: mean and standard deviation of the whole sample; M_0, SD_0: mean and standard deviation for patients without the particular disorder; M_1, SD_1 mean and standard deviation for patients with the particular disorder.
